# Healthy Bed Clothing

**Published:** 1893-03

**Authors:** 


### HEALTHY BED CLOTHING.

In hospital as well as in private practice, great errors are made in the matter of bed clothing for the sick, and particularly for the sick who are suffering from febrile affections. We have got rid of the heavy curtains around the bed; of the grand accumulator of dust and other uncleanliness, the tester; of the heavy vallance which converted the underpart of the bed into a close cupboard, in which all kinds of unwholesome and cumbrous articles lay concealed, including sometimes excreted matter itself ; and we have banished the carpet, which, often a hard-trodden, dust-laden rag, made the floor beneath the bed persistently impure. But the old feather beds, flock mattresses, heavy blankets, thick, impermeable, and dense counterpanes still encumber many a patient, rendering ventilation of his body as impossible as in the days of our forefathers. The thick dense bed and mattress require to be replaced by the light steel elastic bed ; and the clothing under and upon the patient, now so close and heavy, requires to be replaced by clothing that is porous, so that it can be permeated with pure air from without, and can at the same time permit the warm and impure air from the patient to have free exit. The mistake now so generally made lies in the idea that the warmth which the bed calls for is best obtained by close material and close packing. The error is positive. There is nothing that retains warmth in so good and equable a manner as common air at rest. Dense materials cannot keep the body respirably warm. Materials, therefore, both for the bed and for the bed clothing, ought to be porous to a free mechanical extent of porosity. The rule holds good for the clothing of the body in health ; in sickness it is imperative.

